# A Novel Lightweight Real-Time Traffic Sign Detection Integration Framework Based on YOLOv4

**DOI:** 10.3390/e24040487

**Published:** 2022-03-30

**Authors:** Yang Gu, Bingfeng Si

**Affiliations:** School of Traffic and Transportation, Beijing Jiaotong University, Beijing 100044, China; 18114004@bjtu.edu.cn

**Keywords:** intelligent transportation, traffic sign detection, deep learning, lightweight model, feature interaction

## Abstract

As a popular research direction in the field of intelligent transportation, various scholars have widely concerned themselves with traffic sign detection However, there are still some key issues that need to be further solved in order to thoroughly apply related technologies to real scenarios, such as the feature extraction scheme of traffic sign images, the optimal selection of detection methods, and the objective limitations of detection tasks. For the purpose of overcoming these difficulties, this paper proposes a lightweight real-time traffic sign detection integration framework based on YOLO by combining deep learning methods. The framework optimizes the latency concern by reducing the computational overhead of the network, and facilitates information transfer and sharing at diverse levels. While improving the detection efficiency, it ensures a certain degree of generalization and robustness, and enhances the detection performance of traffic signs in objective environments, such as scale and illumination changes. The proposed model is tested and evaluated on real road scene datasets and compared with the current mainstream advanced detection models to verify its effectiveness. In addition, this paper successfully finds a reasonable balance between detection performance and deployment difficulty by effectively reducing the computational cost, which provides a possibility for realistic deployment on edge devices with limited hardware conditions, such as mobile devices and embedded devices. More importantly, the related theories have certain application potential in technology industries such as artificial intelligence or autonomous driving.

## 1. Introduction

The intelligent demand of the automobile manufacturing industry has prompted the vigorous development of related technologies in the fields of autonomous driving and artificial intelligence, and has gradually entered the sophisticated stage of large-scale commercial application over the past few years. In complex road scenarios, traffic facilities can effectively improve the safety performance of vehicles and achieve efficient operation of the road network by providing relevant regulations and guidance information to road users. Traffic facilities normally comprise of traffic signs, traffic signal devices, street lights and lane lines, etc. Relatively speaking, there are varieties of traffic signs, which can intuitively convey specific information, such as guidance and instructions. Therefore, this paper focuses on the detection methods for traffic signs in road scenes.

As one of the joint topics in the fields of intelligent vehicles [1,2], autonomous driving [3,4,5,6] and intelligent transportation systems [7,8,9], traffic sign detection and recognition system (TSDRS) [10,11,12] has received extensive discussions and attention from plenty of experts and scholars in recent years. Common traffic signs are generally composed of symbols, letters, numbers and other elements, which clearly and conspicuously provide road information such as prohibition, warning or guidance in a combined form. With this kind of meaningful information, intelligent vehicles are capable of efficiently perceiving the environmental conditions of road scenes, avoiding obstacles and making decisions in a timely manner, which can greatly reduce the occurrence of accidents. While helping to improve the safety of vehicles, it also facilitates the orderly and smooth flow of the urban road network, making a considerable contribution to the coordinated development of smart cities and smart roads. Traffic sign detection is recognized as a feasible solution that can improve driving safety performance with effect. It exploits computer vision technologies to automatically detect objects of interest in the collected data. As one of the important functions of an automated driving system (ADS) [13] and an advanced driver assistance system (ADAS) [13,14], traffic sign detection has been widely used in the fields of autonomous driving, driver assistance, road network monitoring and maintenance, and traffic scene analysis and research. Hence, it is considered to be one of the remarkable manifestations of the development of automobile intelligence.

Traffic sign detection in real road scenes is susceptible to objective factors, such as occlusion, fouling, illumination changes, motion blur and delay. Conventional traffic sign detection methods are usually based on color or specific shape, the effect of feature extraction is not stable enough, and the detection model has poor adaptability to the environment and insufficient robustness. Traffic sign detection methods based on deep learning are generally derived from the universal object detection frameworks. Even though they have achieved remarkable results in accuracy, the lack of real-time performance makes most of methods difficult to apply to practical detection tasks. In addition, although the lightweight detection framework developed for mobile devices and embedded devices greatly improves the detection speed and reduces the demand for hardware conditions, the performance in terms of accuracy has always been subpar. Therefore, for the complex environment in which traffic signs are located, it is crucial to design a traffic sign detection method with high accuracy, superb real-time performance and strong robustness.

As a typical representative of the end-to-end detection model, the you only look once (YOLO) model has the advantage of being able to predict the categories of objects and generate the bounding boxes simultaneously, which significantly improves the detection efficiency and can be competent for detection tasks with high real-time requirements. Through the continuous improvement of researchers, the performance of YOLO series models has risen steadily. YOLOv2 introduces batch normalization to improve detection accuracy [15]. YOLOv3 deeply refines the feature extraction network and utilizes multiple scale fusion methods for prediction, which effectively improves the detection accuracy while maintaining a high detection speed [16]. YOLOv4 combines the cross stage partial network (CSPNet) in the backbone part, while introducing the spatial pyramid pooling (SPP) and the path aggregation network (PANet) to the neck part, which achieves new heights in both detection accuracy and speed [17].

Nevertheless, the YOLO model takes up more computing resources as the number of network layers deepens, and the resulting latency problem directly affects the timeliness of detection. Meanwhile, the location of traffic signs in the input image is variable, and the size changes quite frequently. The detection model is often sensitive to the resolution of the target, and the small size object only carries a limited number of pixels; thus, the detection performance fluctuates greatly. Furthermore, the performance of the detection model is extremely dependent on the hardware environment. Full-size models are difficult to directly deploy in real-world scenarios, while lightweight models have limited accuracy and cannot meet the basic requirements of some detection tasks. Therefore, finding a suitable balance between performance and efficiency becomes quite a daunting mission. Accordingly, the research work of this paper focuses on the lightweight improvement of YOLOv4, and proposes a lightweight traffic sign detection model YOLO-ML (YOLO-Moderate Lite) with superior real-time performance, acceptable detection accuracy and low hardware requirements. It aims to optimize the latency problem by reducing the computational overhead of the network, enhance the transfer and sharing of information at diverse levels of the input image, and reduce the loss of feature information. It is hoped to improve the detection performance in objective environments, such as scale and illumination changes, while ensuring the detection speed.

The main contributions of this paper are as follows:

(1) According to the fundamental principles of accuracy, real-time, robustness and ease of deployment for traffic sign detection task, we propose a novel lightweight real-time traffic sign detection integration framework based on a deep learning approach. Where it is applied to a real road scene dataset for evaluation and compared with the current mainstream advanced detection models of the same type to verify its effectiveness in complex real-world environments.

(2) By combining and modifying the YOLO and MobileNet network, the efficiency of the feature extraction is optimized, the computational overhead is effectively reduced, and the real-time performance is significantly improved.

(3) A hierarchical feature interaction structure is proposed, which facilitates the fusion and information transfer of multi-scale features. While enhancing the feature processing effect, the model parameters are further reduced to lower the dependence on the hardware environment.

The rest of this paper is organized as follows: Section 2 reviews general object detection frameworks and compares state-of-the-art detection methods for traffic signs; Section 3 introduces our improved ideas and detailed model architecture; Section 4 compares, analyzes and evaluates our proposed traffic sign detection model with other detection models of the same type; Section 5 is the summary of this paper and the prospect of future work.

## 2. Related Work

This section is divided into two parts to introduce the latest research in this field: the first part briefly summarizes the general object detection framework, and the second part focuses on the introduction and analysis of the current advanced detection methods applied to traffic signs.

### 2.1. Object Detection

Object detection usually refers to the process of locating and identifying objects of interest in images or videos, and is one of the basic tasks in the field of computer vision. The research process of object detection algorithms can be roughly divided into two stages: the conventional method period and the deep learning-based method period.

The conventional object detection algorithm generally consists of three steps: feature extraction, region proposal and classification and regression. This type of algorithm usually relies on sliding windows and manually extracted features. The histogram of oriented gradient (HOG) proposed in [18] is calculated on the dense grid of uniformly spaced cells, and overlapping local contrast normalizations are also used to improve performance. Combined with linear support vector machine (SVM) as a classifier, it finally achieves excellent results in the pedestrian detection task. Viola and Jones use Haar-like features to detect faces based on the AdaBoost algorithm, and cascades the obtained strong classifiers to achieve accurate detection results [19]. The scale-invariant feature transform (SIFT) proposed by Lowe, which combines keypoint localization and feature description, is widely used in various fields of computer vision, such as: image recognition, image retrieval, 3D reconstruction, etc. [20]. The deformable part model (DPM) algorithm proposed in [21] adopts the improved HOG feature, combined with the SVM classifier and sliding window, and achieves remarkable results through a multi-component strategy.

Due to the limited ability of manual feature extraction, only relatively low-level features are often extracted. Meanwhile, the process of generating region proposals has a large amount of redundant computation and high computational complexity, the performance of conventional detectors consequently tends to saturate soon. In addition, conventional detection methods still have the defects of difficulty in finding the global optimal solution and poor generalization in complex scenes.

With the substantial improvement of computer hardware computing power, the rapid development of deep learning technology, and the publication of high-quality datasets in recent years, researchers have begun to propose increasingly competitive deep learning-based object detection algorithms. The object detection algorithms based on deep learning automatically extract the hidden features in the image through self-supervised, weakly supervised or unsupervised training methods, and can convert the input pixel data into higher-order and more abstract hierarchical features, which can achieve higher accuracy classification and prediction. At present, the mainstream deep learning-based methods are generally divided into: a two-stage method and a one-stage method. The two-stage method divides the detection problem into two stages: the first stage generates region proposals, which contain approximate location information of objects, and the second stage classifies and regresses the content in the region of interest (ROI). This type of method utilizes explicit region proposals to transform the detection problem into a classification problem of local images within the generated proposal regions. The R-CNN [22] algorithm is the first to successfully apply deep learning technology in the field of object detection. It is characterized by using convolutional neural networks (CNN) to extract the features of the object, and using selective search in the region proposals stage to reduce the amount of computation. Additionally, SVM is used for classification and the object boundary is compensated and corrected with the help of regression algorithm, so as to reduce the deviation of the actual target in the candidate area. There have also been a series of improvements to the original R-CNN algorithm, including: SPP-Net [23], Fast R-CNN [24], Faster R-CNN [25], FPN [26], etc. The one-stage method converts the object detection task into a regression problem, which can directly generate the classification and position coordinates of the target, and the overall process is more concise. Typical methods include: YOLO [27], SSD [28] and RetinaNet [29]. Generally speaking, the two-stage method is more accurate, but the one-stage method has better detection speed and the accuracy is constantly improving. Compared with conventional object detection algorithms, deep learning-based algorithms have the advantages of high speed, good accuracy and strong generalization.

### 2.2. Traffic Sign Detection

Although general object detection method performs well on some benchmark datasets, the task of traffic sign detection is more specific and involves plenty of practical issues, such as: the size of traffic signs is relatively small, the situation that needs to be faced in the road scene is more complicated, and the requirements for detection accuracy and speed are high.

Traffic signs usually have fixed colors and specific shapes that differentiate them from other objects. In order to meet the real-time requirements, Yang et al. [30] propose a fast detection module, which uses the color probability model and HOG features to extract and classify traffic signs. While ensuring the detection accuracy, the speed has also been greatly improved compared with the original method, and the calculation efficiency has been significantly enhanced. Gudigar et al. [31] first used the multi-threshold schemes of the environmental selection strategy to extract the ROI, and then combined the log-polar coordinate mapping of the inner region and the association operation of the reference template to detect traffic signs. Xu et al. [32] propose a traffic sign detection method based on adaptive color threshold segmentation and shape symmetry with statistical hypothesis testing. Firstly, an approximate maximum–minimum normalization method is designed to suppress the interference of high-brightness areas and backgrounds in the image, and then the highlight shape features of the thresholded image are converted into connected domain feature vectors. Finally, experimental results on real datasets demonstrate its excellent robustness in complex environments. Liu et al. [33] propose a traffic sign detection framework based on high contrast region extraction (HCRE) and extended sparse representation classification (ESRC). It only extracts ROIs with high local contrast to strike a balance between detection and extraction rates, and achieves efficient classification of partially occluded traffic signs. Hu et al. [34] propose a general detection framework for traffic signs, vehicles and pedestrians in real-time road environments. The framework consists of dense feature extractors and detectors, and the extracted features are shared with all detectors, which greatly improves the detection speed. Spatial pooling features are introduced as part of aggregating channel features to enhance the robustness of features to noise and image deformation. Incidentally, an object sub-classification method is also proposed in the literature to further improve the generalization performance and demonstrate competitive performance on several benchmark datasets.

The performance of this type of detection method usually depends on the effect of the feature extraction process, and generally requires multiple color features, shape features or mixed features to obtain rich detailed information. Not only that, the detection results are also very susceptible to objective natural factors, such as illumination changes, bad weather and occlusion. As deep learning has achieved surprising results in a lot of fields, related image recognition and detection technologies based on computer vision have also benefited a lot. The deep learning model can automatically extract features, avoiding the limitations of manual feature extraction, and the versatility and robustness of the model are relatively better, which is in sharp contrast with the above methods.

Tabernik and Skocaj [35] propose an improved automatic end-to-end learning detection process based on Mask R-CNN. Through experimental analysis of traffic signs with large intra-category appearance changes, the proposed method achieves no higher than a 3% low error rate. Liu et al. [36] construct an attention-driven bilateral feature pyramid network to learn low-to-high-level foreground semantics, while introducing adaptive receptive field fusion blocks with variable dilation rates to exploit contextual features and suppress occlusion effects. The proposed TSingNet can effectively detect and recognize small and occluded traffic signs in the wild. Kamal et al. [37] detect traffic signs from video sequences by merging the state-of-the-art segmentation architecture SegNet and U-Net. The network uses Tversky loss function constrained by an L1 term and is trained on the CURE-TSD dataset, and the test results on the dataset achieve 95.29% and 89.01% precision and recall. In order to overcome the issues of traffic sign detection in complex backgrounds, such as occlusion and distortion, Li et al. [38] explicitly model the inter-channel interdependence of convolutional features with a small computational cost, and propose an end-to-end saliency traffic sign detection model based on a squeeze-and-excitation-residual network. The network first generates a rough feature map, then uses spatial information and fine details to refine the hierarchical features, and finally fuses the hierarchical feature maps. It exhibits good detection efficiency on all three benchmark datasets. Li and Wang [39] adopt the framework of Faster R-CNN and the structure of MobileNet to design a detector, which uses color and shape information to refine the localization of small traffic signs, and then uses CNN with asymmetric kernel as a classifier. Both the proposed detectors and classifiers show quite superior performance. Zhu et al. [40] propose a text-based cascade traffic sign detection framework with two deep learning components. A fully convolutional network (FCN) is first employed to segment candidate traffic sign regions, providing candidate RoIs, and then another fast neural network is applied to detect text on the RoIs. The proposed method can narrow the search area for text detection and remove texts other than traffic signs, which largely solves the multi-scale problem faced by text detection, and performs well on traffic sign datasets covering both Chinese and English texts. The design in [41,42,43] optimize detection models for the problem that smaller-sized traffic signs occupy fewer pixels than large-scale objects in the input image, which makes detection and classification more difficult, and it achieved excellent detection results.

In summary, the detection method based on deep learning can better segment and detect traffic signs in road scenes for intelligent vehicles, and then provide effective decision support. With the influence of objective conditions such as multi-modality and complex background, the rapid development of intelligent vehicles has continuously put forward higher requirements for traffic sign detection. Therefore, this paper adopts the one-stage method based on deep learning to improve performance, and proposes a traffic sign detection model with the characteristics of lightweight, high precision, low latency and strong robustness.

## 3. Methodology

This paper proposes a traffic sign detection model based on YOLO, which uses diverse task modules to achieve the goal of lightweight improvement, effectively reducing computational overhead and optimizing the latency problem. In the meantime, it enhances the transfer and sharing of cross-level features of the input image and reduces the loss of feature information. It aims to improve the detection performance of traffic signs under objective conditions such as scale transformation and illumination changes while ensuring the detection speed. The method adopts a one-stage multi-frame object detection framework. First, multi-scale features are extracted from the input image through the replaced backbone MobileNet v3 network; then, a feature fusion and redistribution module is constructed before the neck part to provide auxiliary feature mixing operations, so that the information carried by features of different scales is more abundant and balanced; after that, a flexible hierarchical feature transfer strategy is designed, which further enhances the information sharing between deep features and shallow features, and effectively improves the model’s utilization of traffic sign feature information. The overall detection model adopts lightweight network modules, which ensures the accuracy and efficiency of detection tasks while strictly controlling the computational cost.

**Figure 1:**
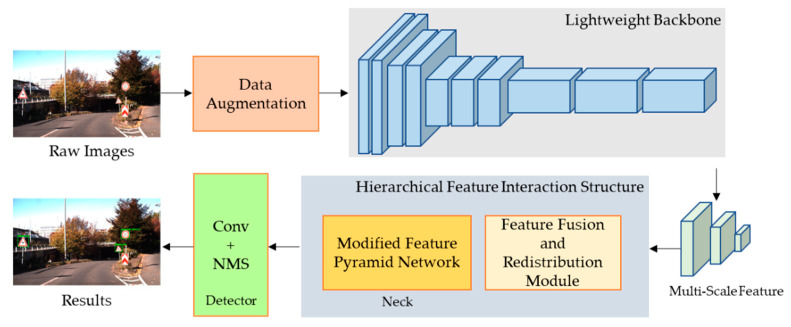
Overall framework workflow for traffic sign detection.

The overview framework workflow for this study is shown in Figure 1.

### 3.1. Overview of YOLOv4

YOLOv4 is the current state-of-the-art one-stage deep learning object detection algorithm, which adopts the theory of regression to directly generate the classification and location information of the target. Compared with other algorithms in the same series, YOLOv4 has made critical improvements in the feature extraction network and multi-feature fusion, and the accuracy and real-time performance have been significantly enhanced. The architecture of YOLOv4 is mainly composed of backbone, neck and head.

Specifically, the backbone firstly applies the CSPDarknet53 network, the fundamental of which is to continuously copy the feature maps of the base layer, and then use the dense blocks to pass them to the next stage. One feature map is divided into two parts, one part is subjected to convolution operation, and the other part is subjected to concatenate operation with the result of convolution. The change in gradient is integrated into the feature map, which reduces the overhead of inference computation and the cost of memory to a certain extent. The Mish activation function [44] used by YOLOv4 is a self-regularized non-monotonic neural activation function, which has a more remarkable boost to the performance of deep networks and is easier to implement than other common activation functions.

**Figure 2:**
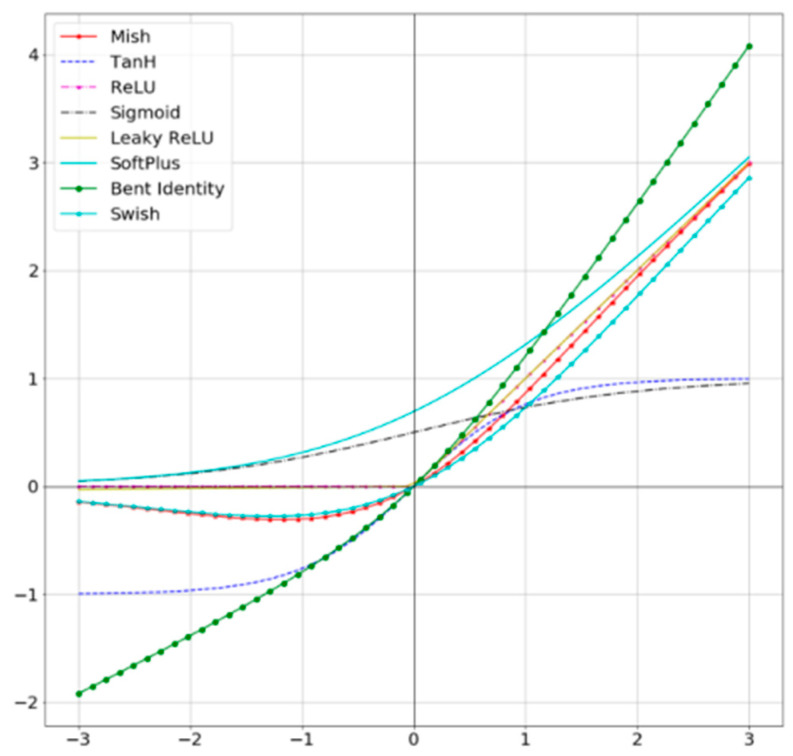
Comparison of common activation functions.

Figure 2 shows a comparison of common activation functions, from which it can be seen that the non-monotonic property does not completely truncate negative values but allows relatively small negative inputs to be preserved as negative outputs. The flow of information promotes smoothness at each point and makes gradient descent more effective, resulting in finer accuracy and generalization. The formula is as follows:

(1) $$Mish=x*tanh\left(ln\left(1+e^{x}\right)\right)$$

Secondly, the neck part mainly consists of a series of network layers that mix and combine image features, and pass the image features to the prediction layer. The integrated SPP module is able to notably increase the receptive field, isolate vital contextual features, and only have a minimal impact on network speed. The process of aggregating features mainly relies on the PANet structure. By introducing a shortcut path, the bottom-up propagation efficiency is improved, so that the top layer can also obtain fine-grained local information, which strengthens the effect of feature fusion.

**Figure 3:**
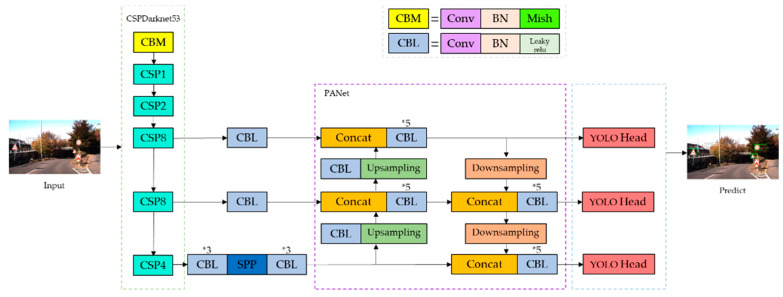
Structural block diagram of YOLOv4.

Finally, the head part responsible for detection adopts the anchor-based method. It applies anchor boxes on feature maps and generates final output vectors with class probabilities, object scores and bounding boxes. Figure 3 provides the structural block diagram of YOLOv4.

**Figure 4:**
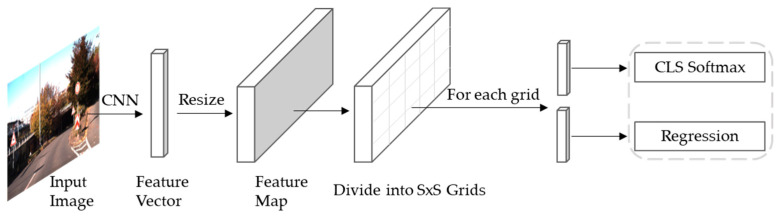
Schematic flow diagram of YOLOv4.

The object detection process of YOLOv4 is to first resize the raw image to a specific resolution size as input. The input image is divided into S×S grids according to the multi-scale feature map, and each grid is responsible for detecting objects whose centers fall in the grid. Confidence scores are generated by predicting several bounding boxes and the probability of the class. Confidence scores reflect the degree of overlap between the bounding box and the ground truth box. At last, the non-maximum suppression (NMS) algorithm is applied to extract the most likely objects and their corresponding bounding boxes from the output results. Figure 4 presents the schematic flow diagram of YOLOv4.

### 3.2. Lightweight Backbone

Even though YOLOv4 adopts the CSPNet architecture in the backbone to help enrich the gradient combination information, the one-stage detection method has verified its effectiveness in many scenarios. However, for intelligent vehicles, more attention is paid to efficient architecture, low latency and easy to meet the requirements of embedded or edge devices. The original backbone network has a large number of layers, and the high computational complexity results in extensive computing resources, which makes it difficult to directly deploy on real devices. In order to improve the above problems, this paper uses an efficient lightweight network to replace the original backbone of YOLOv4 in the feature extraction stage, so as to design a stable detection model that is more in line with the actual traffic sign detection requirements. Compared with full-size networks, the main features of lightweight networks are fewer model parameters, low computational overhead, and short inference time. It is widely used in scenarios where storage conditions and hardware power consumption are limited, such as mobile devices and embedded devices.

MobileNet mainly focuses on optimizing the latency problem, using the idea of depthwise separable convolution to reconstruct the network. It replaces standard convolution with depthwise convolution and pointwise convolution, which greatly reduces the number of parameters and computation of the network.

**Figure 5:**
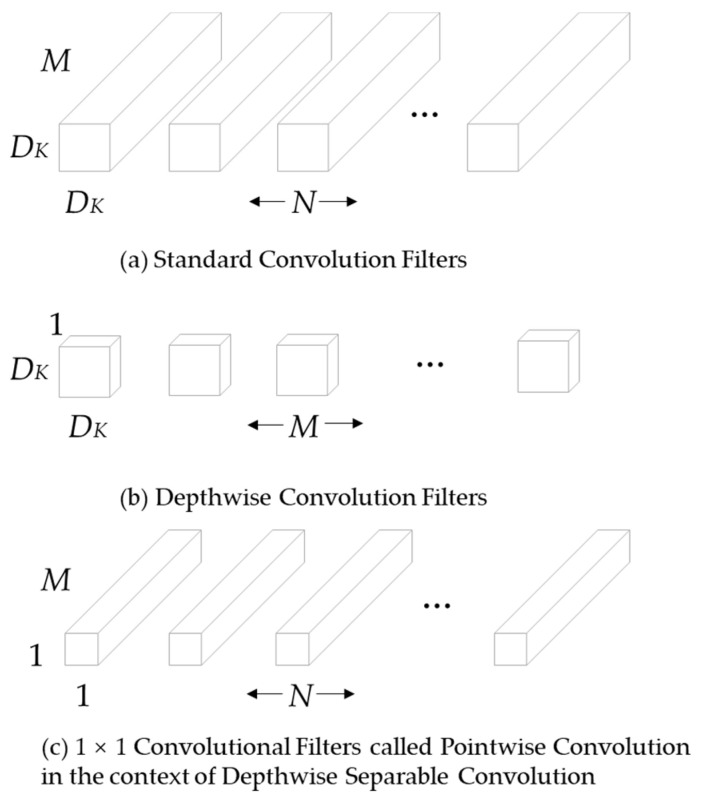
The standard convolutional filters are replaced by depthwise convolution and pointwise convolution to build a depthwise separable filter.

The input feature map F dimension is $\left(D_{F},D_{F},M\right)$, where $D_{F}$ is the spatial width and spatial height of the input features, and M is the number of input channels. The dimension of the output feature map G is $\left(D_{G},D_{G},N\right)$, where $D_{G}$ is the spatial width and spatial height of the output feature, and N is the number of output channels. The size of the standard convolution K used is $\left(D_{K},D_{K},M,N\right)$ as shown in Figure 5a, where $D_{K}$ is the spatial dimension. The convolution calculation formula of standard convolution is:

(2) $$G_{k,l,n}=\sum_{i,j,m}K_{i,j,m,n}\cdot F_{k+i-1,l+j-1,m}$$

The corresponding calculation amount is: $D_{K}\cdot D_{K}\cdot M\cdot N\cdot D_{F}\cdot D_{F}$.

After the standard convolution $\left(D_{K},D_{K},M,N\right)$ is replaced by depthwise convolution and pointwise convolution: the depthwise convolution is responsible for filtering, the size is $\left(D_{K},D_{K},1,M\right)$ as shown in Figure 5b, and the output feature is $\left(D_{G},D_{G},M\right)$; the pointwise convolution is responsible for channel conversion, and the size is (1,1,M,N) as shown in Figure 5c, and the final output is $\left(D_{G},D_{G},N\right)$. The convolution formula for depthwise convolution is:

(3) $${\hat{G}}_{k,l,n}=\sum_{i,j}{\hat{K}}_{i,j,m,}\cdot F_{k+i-1,l+j-1,m}$$

Among them, $\hat{K}$ is the depthwise convolution, and the convolution kernel is $\left(D_{K},D_{K},1,M\right)$, where mth convolution kernels are applied on the mth channel in F, producing the mth channel output on $\hat{G}$. The computational cost of depthwise convolution and pointwise convolution is: $D_{K}\cdot D_{K}\cdot M\cdot D_{F}\cdot D_{F}+M\cdot N\cdot D_{F}\cdot D_{F}$. The amount of computation is reduced:

(4) $$\frac{D_{K}\cdot D_{K}\cdot M\cdot D_{F}\cdot D_{F}+M\cdot N\cdot D_{F}\cdot D_{F}}{D_{K}\cdot D_{K}\cdot M\cdot N\cdot D_{F}\cdot D_{F}}=\frac{1}{N}+\frac{1}{{D^{2}}_{K}}$$

**Figure 6:**
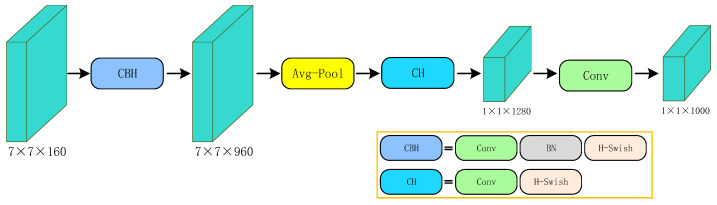
Efficient last stage.

After the accumulation of the previous two generations, MobileNet v3 [45] has become a star in the lightweight network field with excellent performance and speed. It improves the inference speed of the network by redesigning the expensive time-consuming layer structure. First, the number of initial filters is reduced from 32 to 16, which reduces inference time and computation while maintaining the original accuracy. Furthermore, the original last layer of the network uses 1 × 1 convolution to expand the features into a high-dimensional space to generate final features, which helps enrich the predicted features, but at the cost of additional latency. MobileNet v3 optimizes the interaction of the network layers in the last stage, replacing the previous 7 × 7 spatial resolution with 1 × 1 spatial resolution for computation, which greatly reduces latency and preserves high-dimensional features. In addition, by removing the front and back connections of the previous bottleneck layer, the computational complexity is further reduced, effectively decreasing the inference time by 11% and refining the parameter redundancy without loss of performance. Figure 6 displays the improved efficient last stage architecture.

**Figure 7:**
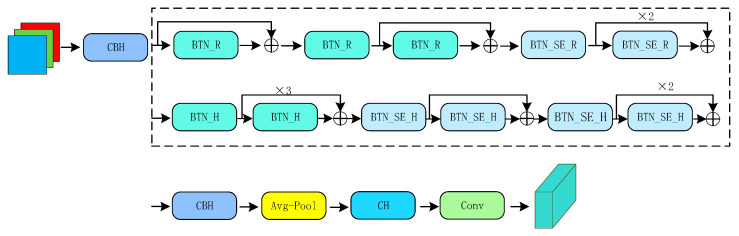
MobileNet v3 architecture. SE denotes whether there is a Squeeze and Excite in that block. BTN denotes bottleneck, H denotes h-swish and R denotes ReLU.

MobileNet v3 inherits the depthwise separable convolution and linear bottleneck residual structure of the previous generation, applies the Squeeze and Excite (SE) channel attention structure in the residual layer, and proposes a new activation function h-swish and then uses differentiated activation functions according to different layers to reduce computational overhead costs. By replacing the SE bottleneck with a fixed size, the accuracy is effectively improved at the cost of only a modest increase in the number of parameters, and without a significant increase in latency. It is worth mentioning that in a series of variants defined by MobileNet v3, in order to ensure the accuracy of feature extraction, this paper adopts the MobileNet v3 Large version. It can be seen from the above that the backbone we attempt to replace can greatly reduce the amount of computation, and effectively realize the lightweight of the detection model while ensuring considerable accuracy. Figure 7 shows the replaced lightweight backbone MobileNet v3.

### 3.3. Hierarchical Feature Interaction Structure

The deep network has a large receptive field and a strong ability to represent semantic information, but the feature map resolution is low, and the spatial geometric features lack details. On the other hand, although the shallow network has strong geometric information representation ability and high resolution, it lacks semantic information representation ability and has a small receptive field. Early object detection methods mainly rely on a single high-level feature, but these methods have obvious defects, that is, small objects themselves contain less pixel information, which is easily lost in the process of downsampling. The deep small-scale feature map cannot provide sufficient resolution information after high-fold downsampling, and is only suitable for processing the detection of larger-sized objects. Small object detection still needs to rely on large-scale feature maps. The classical image pyramid method is computationally expensive, and in order to control the computational cost and efficiently propagate deep features, the feature pyramid structure is proposed [26].

The interaction of features in the original YOLOv4 network is a PANet structure that combines top-down and bottom-up methods. Although the increase in shortcut connections shortens the information path between shallow features and deep features and accelerates information fusion, the efficiency of non-adjacent information propagation between deep features and shallow features is still limited. In addition, the shape of traffic signs is relatively fixed, and the PANet structure is slightly cumbersome for the task of traffic sign detection. Considering the real scene where the intelligent vehicle is located, the size of the original image collected by the on-board camera is generally large, the position of traffic sign in the collected image is obviously different, and the size changes frequently. For the purpose of further optimizing the interaction of deep and shallow feature information, improving the detection accuracy of target objects and reducing network structure redundancy, this paper proposes a novel feature interaction structure inspired by the feature fusion mechanism and FPN theory. The structural design is mainly composed of a feature fusion and redistribution module and an improved feature pyramid, which flexibly transfers multi-scale features without significantly increasing additional computational cost, so that the model has reliable feature expression ability and lightweight execution effect.

#### 3.3.1. Feature Fusion and Redistribution Module

The feature fusion and redistribution (FFR) module is a lightweight feature processing module, which is mainly responsible for receiving the multi-scale features extracted by the backbone network, connecting the feature pyramid and providing it with feature input. The original multi-scale features are reassigned back to the corresponding scale after fusion, in order to improve the effect of feature utilization and enhance the performance of detection accuracy.

The features in YOLOv4 adopt the conventional layer-by-layer propagation structure, which mainly focuses on the feature information of adjacent scales, which may miss a large quantity of effective combination pairs, and the information propagation efficiency between non-adjacent or far-distant features is throughout limited. In fact, reasonably integrating high-level semantic information and low-level location information and correlating features at each level is one of the best ways to improve the accuracy of the detection model. Therefore, the design idea of the FFR module is to provide an auxiliary feature mixing operation before passing the multi-scale features to the finer feature aggregation method, so that the information carried by the features of different scales is richer and more balanced.

**Figure 8:**
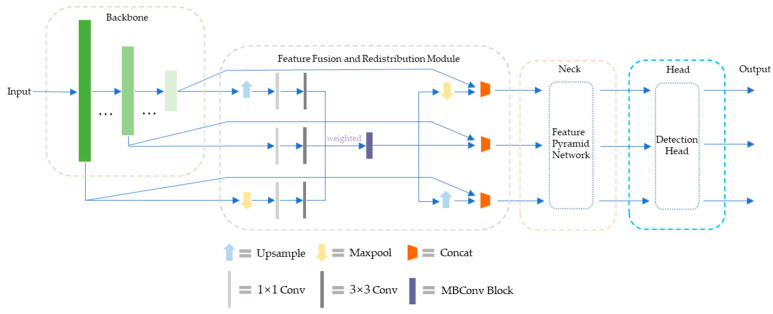
The architecture of feature fusion and redistribution module.

Specifically, feature maps of different scales have different semantic information. The FFR module fuses multi-scale features between different layers in the backbone network, allowing redistribution and direct connection of raw features, which is conducive to the multi-source transmission of context information in the lightweight network, while avoiding high computational overhead and introducing numerous parameters. The original features extracted by the backbone are passed through a single 1 × 1 convolution, and each feature map is linearly combined on different channels. Then, the 3 × 3 separable convolution operation is used to obtain more feature information of width and depth, which can greatly reduce the computational complexity and improve the execution speed. The number of input feature maps of the module and the number of output feature maps do not need to be completely equal. The input feature map coordinates the proportion of features at different scales by introducing additional weights, makes full use of high and low-level features with different granularities, and adaptively learns the importance of features at different scales to the detector by training the network. The fused feature map passes through a MobileNet convolution block (MBConv) and is then redistributed back to the corresponding scales. MBConv uses 7 × 7 kernel to replace the common 3 × 3 kernel to increase the receptive field of the fusion feature layer, which is beneficial to improve the accuracy of the detection model, and the efficiency of MBConv with large size kernel is higher. At last, the output features of these branches are concatenated with the original-scale feature maps, and the generated sub-feature blocks are input to the neck part. During this process, upsampling and maxpooling operations are mainly responsible for matching between feature maps of diverse resolutions. The architecture of the feature fusion and redistribution module is provided in Figure 8.

Eventually, the multi-scale features from the backbone network are fused and optimally allocated to effectively ensure the transfer of cross-level information, helping to improve the detection accuracy of targets of different sizes without significantly increasing the latency cost.

#### 3.3.2. Multi-Scale Feature Transfer Strategy

Conventional FPN fuses feature maps from top to bottom through coupling, while PANet further improves the effect of feature fusion by using bidirectional feature propagation. In order to deeply optimize the interaction and sharing of deep and shallow network features, the redundancy of some complex structures is reduced, and the accuracy and robustness of the detection model are improved, especially the detection performance of small-sized traffic signs. We designed a novel feature enhancement mechanism by combining the characteristics of the traffic sign detection task in real-world scenarios. Different from the conventional layer-by-layer propagation structure, after the features are effectively shared and fused, the shallow features can no longer only distinguish relatively simple objects, and the deep features are no longer mainly responsible for distinguishing complex objects. Compared with the conventional mechanism, this method can effectively strengthen the features at all levels from space to semantics, and the multi-dimensional information provided by the fused deep and shallow features is able to become more comprehensive. By transferring and sharing the receptive field content of different scales, the detection model learns and perceives the rich details and location information of the target to be detected, so as to obtain clearer and more accurate features.

**Figure 9:**
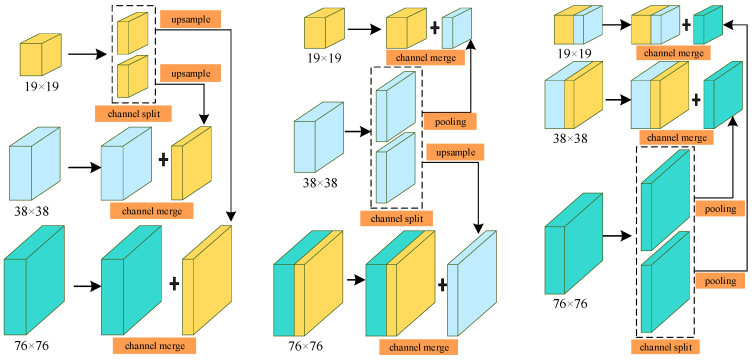
Schematic diagram of multi-scale feature fusion and sharing.

Specifically, after the features of each scale are split by channels, upsampling or the maxpool operation is used to match the scales of other features, and the generated intermediate features are shared with each other, and finally integrated into the fusion features of the three scales. The multi-scale feature transfer strategy divides the 19 × 19, 38 × 38 and 76 × 76 feature maps extracted from the backbone part into small-scale features, medium-scale features and large-scale features. As shown in the Figure 9 below, these features are repeatedly fused and shared, effectively balancing the information carried by features at different scales and realizing the mutual transfer and sharing of high-level semantics and shallow features, thereby providing more accurate detection effects. The detailed steps are as follows:

Step 1: The 19 × 19 small-scale features are divided into two parts in the channel dimension. One part is fused with medium-scale features after upsampling to generate 38 × 38 intermediate features, and the other part is high-fold upsampling to match large-scale features to generate 76 × 76 intermediate features.

Step 2: After the medium-scale features are divided into two parts according to the channel dimension, one part is fused with small-scale features after maxpool to generate 19 × 19 intermediate features, and the other part is upsampled and fused with the 76 × 76 intermediate features generated in step 1 to form large-scale feature blocks.

Step 3: After the large-scale features are split by channels, one part is fused with the 38 × 38 intermediate features generated in step 1 after maxpool to form a mesoscale feature block, and the other part is fused with the 19 × 19 intermediate features generated in step 2 to form a small-scale feature block.

Eventually, the features of each scale will be distributed to the features of other scales to enhance the information carried by each other.

## 4. Experimental Results

### 4.1. Benchmark and Data Preprocessing

Considering that the related research results of traffic sign detection ultimately need to be applied in real road scenes and meet the needs of real-time detection tasks, this paper uses the German Traffic Sign Detection Benchmark (GTSDB) [46] dataset to conduct experiments and analyze the experimental results. The original road scene images in the GTSDB dataset are extracted from real, complete video sequences with strong continuity. This is also one of the important features that distinguish it from some other datasets. The image data collected by the panoramic camera have poor correlation and the traffic scene is relatively clean, which makes the model training process lack of some disturbing negative samples such as billboards, pedestrians, vehicles, traffic lights, etc. In addition, the raw image data in the GTSDB dataset also contain scenes of severe weather such as foggy or rainy days and the interference of objective factors such as motion blur, which make the image data in the dataset more reflective of the complex road conditions in reality. The difficulty for the detection model to deal with is thus increased, and the detection task becomes more challenging.

**Figure 10:**
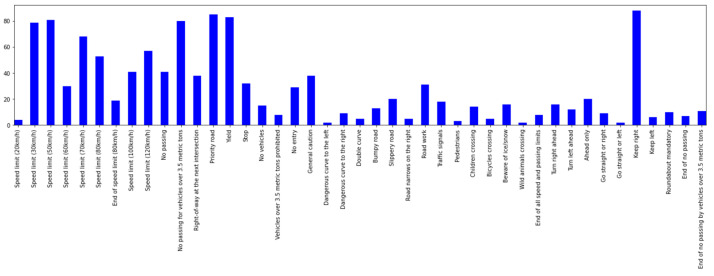
Data and class distribution.

The original dataset contains nine hundred road images with traffic signs from Germany and signs have forty-three labelled classes. We randomly select 600 images as the training set, and the remaining 300 images are used as test images to check the performance of the model. Figure 10 shows the original distribution of images in the dataset, the horizontal axis denotes different label types, and the vertical axis denotes the number of corresponding traffic signs.

According to the different attributes of these traffic signs, they can also be grouped into four categories such as mandatory, danger, prohibitory and other. Table 1 shows example images of some traffic signs in four categories, respectively. It can be seen that some traffic signs with a circular shape and blue ground characteristics can be classified as mandatory; some traffic signs with red border, triangular shape and white ground characteristics can be classified as danger; some traffic signs with red border, circular shape and white ground characteristics can be classified as prohibitory; the remaining traffic signs are classified as other.

**Figure 11:**
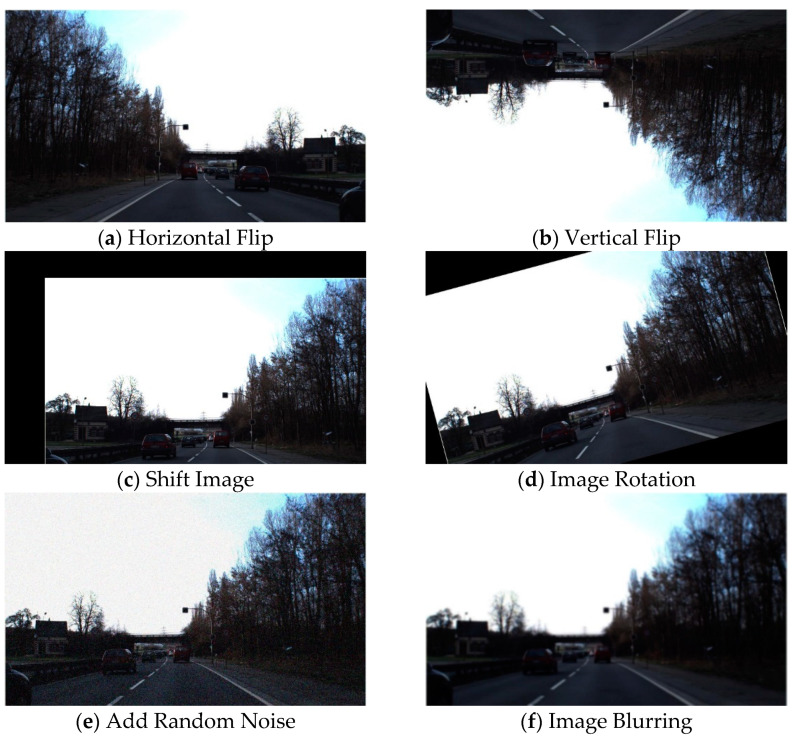
Image example after data augmentation.

Due to the relatively unbalanced distribution of raw data in the dataset, the number of traffic signs in some categories is less than other categories. Hence, we use data augmentation in the preprocessing stage to expand the number of training set samples and optimize the stability of model training. Specifically, these augmentations include the methods of horizontal flip, vertical flip, add random noise, image rotation, shift image pixels in x and y directions, and image blurring, increasing the number of original training images by a factor of six. Some of the effects are shown in Figure 11 below:

### 4.2. Experimental Environment and Evaluation Metrics

The training process of the YOLO-ML model is implemented based on the Google Colab platform, which combines the idea of transfer learning. We first use the weight file pre-trained in the MS COCO dataset for training, and then transfer to the GTSDB dataset and perform fine tuning. During this process, the initial learning rate for model training is set to 0.001, the batch-size is set to 64, and the number of iterations is 8000. The test experiments are run on a local device with 16 GB of memory, an Intel Core i7-7700K for the CPU, and an NVIDIA GTX 1080 Ti with 3584 CUDA cores and 11 GB of video memory for the GPU.

In order to determine the effectiveness and stability of the proposed YOLO-ML model for traffic sign detection in complex road scenes, several sets of experiments are set up to verify and evaluate its real performance. We use a variety of evaluation metrics to examine the performance of our model in terms of accuracy, real-time and lightweight, and compare and analyze the results with other advanced detection models of the same type.

In terms of accuracy, mean average precision (mAP) is usually used as a metric to quantitatively evaluate the overall accuracy of the detection model, while average precision (AP) is used to measure the accuracy of detecting a specific type of target. AP represents the area under the precision and recall curves, and mAP is the result of averaging the AP values of different categories in the dataset. Precision evaluates the accuracy of model detection and is the number of correctly predicted positive samples divided by the total number of predicted positive samples. Recall evaluates the ability of the model to find all positive samples, which is the number of correctly predicted positive samples in the total number of all positive samples. True positive (TP) indicates that the detected traffic sign exactly matches the true meaning of the traffic sign; false positive (FP) indicates that although a traffic sign is detected, the detection result does not match the true meaning of the traffic sign; false negative (FN) indicates a traffic sign that the model fails to detect. Table 2 shows the details of the relevant evaluation metrics.

In terms of real-time performance, we count inference time and frames per second (FPS) to evaluate the detection speed of the model. Inference time indicates the average processing time from input to output for a single image in the test set. FPS mainly refers to the number of frames transmitted per second in the image field. The more frames per second, the smoother the motion displayed.

In terms of lightweight, this paper focuses on exploring the construction of a lightweight traffic sign detection model, thereby reducing the demand for computing power of hardware devices and optimizing the occupation of computing resources. Therefore, this paper counts the number of parameters and floating-point operations (FLOPs) of different detection models to compare the size and computation to evaluate the lightweight degree of the YOLO-ML model. FLOPs indicate the number of floating-point operations required from the input to the output of the model, which reflects the complexity of the detection model to a certain extent.

### 4.3. Visualization of Detection Results

This subsection details the detection results of the proposed YOLO-ML model applied to traffic signs appearing in real road scenes in the GTSDB dataset. As we all know, although traffic signs have a relatively fixed shape compared to ordinary objects, the detection process of traffic signs is usually completed under non-static conditions in reality, and stable detection performance needs to be guaranteed under various natural conditions, which makes the task of traffic sign detection greatly challenging. Thus, this paper selects several representative detection results from the experimental results of real road scenes for display.

**Figure 12:**
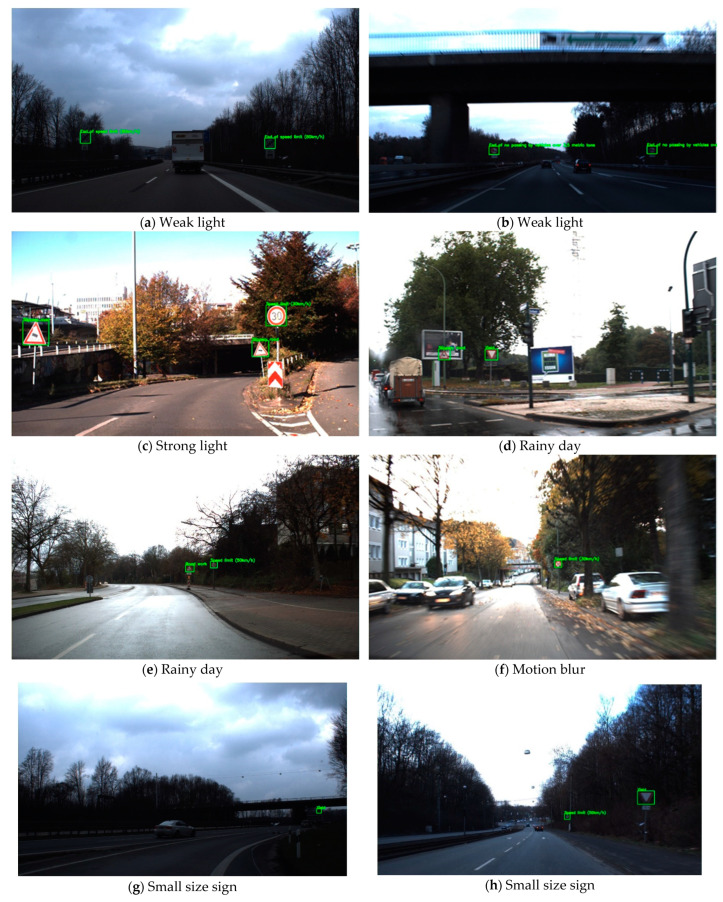
Detection results of German Traffic Sign Detection Benchmark (GTSDB) dataset.

Figure 12 selects representative experimental results in different realistic environments, such as low-light environment, strong-light environment, rainy weather, motion blur, and small objects to be detected in the distance. Firstly, by comparing Figure 11a–c, it can be found that the proposed YOLO-ML model is not sensitive to changes in the intensity of illumination, and its performance is quite stable in both low-light and strong-light environments, which is enough to demonstrate that the model can cope with the detection task in the illumination changing scene with ease.

Secondly, the vehicle inevitably encounters blurred images during driving, such as movement, bad weather and other factors that may cause the images to be unclear. Figure 12d–f show the detection results in rainy weather and motion blurred states, respectively. As can be seen from the figure, our model is fully capable of performing detection tasks in a rainy environment, and can precisely detect traffic signs even when the image captured by the camera is blurred due to vehicle motion. It is manifested that our model has good adaptability to weather changing scenes under non-extreme weather conditions, and has a satisfactory performance in dealing with the interference of objective factors, such as image blur that is unavoidable in reality.

In addition, this paper also considers the issue that the size of distant traffic signs in images collected by intelligent vehicle cameras is generally small in real scenarios. As can be seen from Figure 12g,h, the detection results of small-sized traffic sign objects are still clear and precise even under the condition of relatively insufficient light in the distance. The bounding box is not biased at all and the traffic sign classification results are also very accurate. The bounding box has no deviation and the traffic sign classification results are also surprisingly accurate. Through continuous observation, we find that although a few distant traffic signs have accurate detection positions but the classification results are wrong, the model can also re-provide correct classification results with slightly closer images extracted from the next few frames of a continuous video sequence. It demonstrates that the proposed YOLO-ML model also has a considerable performance in dealing with the difficult problem of small object detection.

Through a series of detection results, it can be found that the bounding box position of the detected traffic sign is accurate, and the classification result to which the traffic sign belongs is also completely correct. Hence, it is fully demonstrated that the traffic sign detection model proposed in this paper not only has reliable accuracy, but also can cope with the detection requirements in complex real-world environments, which manifests that the model has good robustness and adaptability. As a matter of fact, it can be seen from the above several sets of visualization experiment results in different environments that the detection model proposed in this paper fully takes into account the characteristics of the traffic sign detection task, and strengthens the interaction between hierarchical networks and the effectiveness of feature fusion. Our detection model can accurately identify and locate distant small targets, which also proves that the feature fusion and interaction strategy in this paper is meaningful.

### 4.4. Performance Comparison

#### 4.4.1. Quantitative Analysis

In order to objectively evaluate the detection performance of the YOLO-ML model in the real outdoor road scene, the experiment adopts a variety of evaluation metrics from different angles for quantitative comprehensive evaluation. This subsection compares the proposed model with state-of-the-art full-size models Faster R-CNN, YOLOv3 and YOLOv4, and lightweight models SSD, YOLOv3-Tiny and YOLOv4-Tiny. The comparison experiments and ablation studies are performed on the same local hardware.

Firstly, in terms of accuracy, Table 3 shows the detection results of the proposed model and other mainstream detection models on the GTSDB dataset. It can be seen from the table that the overall detection accuracy of the YOLO-ML model on the GTSDB test set reaches 80.62%, which is significantly better than the three typical lightweight models of SSD, YOLOv3-Tiny and YOLOv4-Tiny. Compared with the lightweight versions YOLOv3-Tiny and YOLOv4-Tiny derived from the YOLO series of models, the mAP of the YOLO-ML model proposed in this paper is 14 and 8 higher than them, respectively, which is almost close to the accuracy of the full-size model YOLOv3. In addition, the YOLO-ML model also has an advantage in the average precision of traffic signs in different categories in the GTSDB dataset.

**Figure 13:**
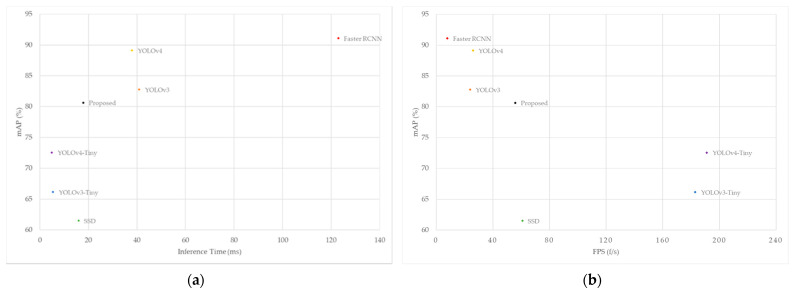
Detection speed comparison of different models on the GTSDB dataset: (**a**) inference time performance and (**b**) FPS performance.

Secondly, inference time and FPS are major metrics to examine the detection speed and real-time performance of the detection model. Figure 13 plots the inference time and FPS of different detection models. The horizontal axis corresponds to the inference time and FPS, respectively, and the vertical axis is the mAP of the GTSDB dataset. As can be seen from Figure 13, the FPS of the YOLO-ML model proposed in this paper reaches 57, which is higher than the 26 FPS of YOLOv4, and the average inference time for a single image is also shortened by half, from 38.72 ms of YOLOv4 to 17.66 ms. In terms of real-time performance, the YOLO-ML model is close to the SSD model, which is known for its excellent detection speed, only 2 ms slower than the average inference time of the SSD model. The remarkable execution speed of our model is enough to demonstrate that it can effectively take into account the simultaneous improvement of accuracy and speed, and can meet the needs of real-time detection tasks to a certain extent.

In addition, in order to compare the lightweight degree of different models, we use analysis tools to count the number of parameters and FLOPs of each model, and Table 4 shows the comparison results. It is worth noting that there is no specific relationship between the number of parameters, FLOPs and detection efficiency. Although some models have more parameters or a larger amount of computation, they can still achieve better detection speed. In comparison, the lightweight detection models have obvious advantages in terms of the number of parameters and control of computational overhead. Compared with the original YOLOv4 model, the model proposed in this paper remarkably simplifies the number of parameters, and also greatly saves the amount of computation, which reflects that the strategy of replacing lighter backbones is fairly effective. In general, our model has obvious advantages over the full-size models in terms of the number of parameters and the amount of computation, and far outperforms other lightweight models in terms of accuracy.

#### 4.4.2. Ablation Study

In order to verify the effect improvement brought by the different methods introduced in the YOLO-ML model, we conducted ablation experiments to evaluate the effectiveness of data augmentation, backbone replacement and hierarchical feature interaction structure.

Considering that the original training images of the GTSDB dataset are only 600 and the class distribution is relatively uneven, we used data augmentation to increase the original training images by a factor of 6 to 4200. It can be seen from Table 5 that after data augmentation, the generalization of the original YOLOv4 model is improved, and the mAP is increased from 85.34 to 89.11, which indicates the necessity of data augmentation operation. After the backbone of the original YOLOv4 model is replaced, the number of parameters and computational complexity is effectively reduced, and a lot of detection accuracy is also sacrificed. The hierarchical feature interaction structure proposed in Section 3.3 is still essentially a modified feature pyramid structure, and the mAP of the model is increased from 78.20 to 80.62, which improves the detection accuracy to a certain extent. Moreover, we further optimized the number of parameters and reduced the computational overhead, so that the detection model is simplified while still maintaining high accuracy. In terms of real-time performance, the FPS is increased from 26 to 57, which obviously surpasses all full-size models.

In summary, compared with the original model YOLOv4, the number of parameters of the YOLO-ML model proposed in this paper is only 15.8%, and the FLOPs are also reduced to 27.8%. Relatively speaking, our model is more friendly to the requirements of hardware conditions, and it is believed that it may have more advantages under the condition of limited equipment environment.

## 5. Conclusions

In this paper, an effective lightweight real-time traffic sign detection model YOLO-ML is proposed to address the practical problems of high latency, difficult implementation, and limited real-time performance in current traffic sign detection methods. The core advantages of our model mainly include: rapid detection speed, considerable accuracy, small size, and low hardware requirements. It can be said that a reasonable balance between detection performance and efficiency has been successfully found. First, the speed of feature extraction is improved by optimizing the backbone network, and the computational overhead is effectively reduced, which not only alleviates the latency issue but also significantly refines the real-time performance of the detection model. Then, a hierarchical feature interaction structure is proposed for the conventional layer-by-layer feature transfer mechanism, which enhances the transfer and sharing of information between layers, reduces the loss of feature information, and facilitates the fusion and information transfer of multi-scale features. Moreover, the parameter redundancy is further decreased to compress the model size, so that the demand for hardware is reduced. It aims to heighten the detection performance of traffic signs under changes in objective conditions such as scale and illumination while ensuring the detection speed. The experiment is carried out based on the real road dataset GTSDB. By comparing with the current mainstream advanced traffic sign detection models of the same type, it is verified that the YOLO-ML model has superior detection speed, considerable detection accuracy, and small model size. In addition, the YOLO-ML model is not sensitive to the changes in scene illumination, and has good detection results for small size traffic signs at the far end, which reflects the generalization and robustness, and the potential to be applied in real road scenes to a certain extent. In the future, we will further optimize the performance and stability of our detection model, and attempt to transfer it to other work scenarios to fulfill the detection task. Last but not least, we are going to turn our attention to how to process detection tasks with large amounts of data in real time; how to cooperate with other sensors to maximize the safety of autonomous driving; and how to realize cross-platform portable deployment, etc. It is believed that in the future, object detection for real road scenes is still a topic worthy of long-term research.

## Figures and Tables

**Table 1:** Examples of different categories traffic signs in GTSDB dataset.

Mandatory		Danger		Prohibitory		Other	
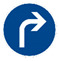	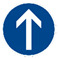	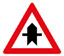	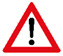	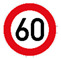	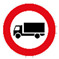	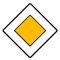	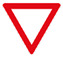
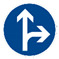	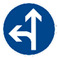	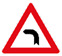	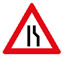	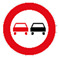	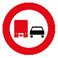	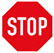	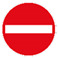
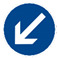	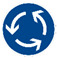	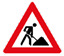	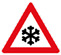	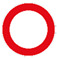		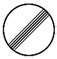	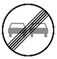

**Table 2:** Details of accuracy metrics.

Metrics	Formula	Domain	Reference
Precision	Precision=TP/(TP+FP)	[0,1]	[47]
Recall	Recall=TP/(TP+FN)	[0,1]	[47]
AP	$AP=\sum_{i=1}^{N}Pinterp(r)$	[0,1]	[48]
mAP	$mAP=\frac{1}{N}\sum_{i=1}^{N}APi$	[0,1]	[48]

**Table 3:** Accuracy comparison of four categories traffic signs in GTSDB dataset obtained by different detection models.

Methods	Average Precision				mAP
	Mandatory	Danger	Prohibitory	Other	
Faster R-CNN	88.12	93.02	94.88	88.29	91.08
YOLOv3	73.92	85.89	87.61	83.56	82.75
YOLOv4	82.41	91.63	92.83	89.56	89.11
SSD	52.78	64.95	66.32	61.85	61.48
YOLOv3-Tiny	57.60	64.69	73.83	68.31	66.11
YOLOv4-Tiny	65.69	73.65	81.36	69.43	72.53
Proposed	74.43	81.92	84.79	81.35	80.62

**Table 4:** Comparison of model parameters and FLOPs.

Model	Backbone	Input Resolution	Params (M)	FLOPs (G)	mAP
Faster R-CNN	Resnet 50	800 × 600	43.3	533.6	91.08
YOLOv3	Darknet-53	608 × 608	61.6	65.4	82.75
YOLOv4	CSPDarknet-53	608 × 608	64.0	59.7	89.11
SSD	MobileNet v1	300 × 300	5.57	2.3	61.48
YOLOv3-Tiny	Darknet	608 × 608	8.7	5.5	66.11
YOLOv4-Tiny	CSPdarknet53-tiny	608 × 608	5.9	6.8	72.53
Proposed	MobileNet v3	608 × 608	10.1	16.6	80.62

**Table 5:** Ablation study results.

Methods	mAP	Params (M)	FLOPs (G)	FPS
YOLOv4 without data aug	85.34	64.0	59.7	26
YOLOv4 + data aug	89.11	64.0	59.7	26
YOLOv4 + data aug + MobileNet v3	78.20	27.2	23.0	52
YOLOv4 + data aug + MobileNet v3 + HFI	80.62	10.1	16.6	57
